# Neonatal annulus fibrosus regeneration occurs via recruitment and proliferation of *Scleraxis*-lineage cells

**DOI:** 10.1038/s41536-019-0085-4

**Published:** 2019-12-20

**Authors:** Olivia M. Torre, Victoria Mroz, Anthony R. Martinez Benitez, Alice H. Huang, James C. Iatridis

**Affiliations:** Leni & Peter W. May Department of Orthopaedics, Icahn School of Medicine at Mount Sinai, One Gustave Levy Place, Box 1188, New York, NY 10029-6574 USA

**Keywords:** Regeneration, Regenerative medicine

## Abstract

Intervertebral disc (IVD) injuries are a cause of degenerative changes in adults which can lead to back pain, a leading cause of disability. We developed a model of neonatal IVD regeneration with full functional restoration and investigate the cellular dynamics underlying this unique healing response. We employed genetic lineage tracing in mice using *Scleraxis* (*Scx*) and *Sonic hedgehog* (*Shh*) to fate-map annulus fibrosus (AF) and nucleus pulposus (NP) cells, respectively. Results indicate functional AF regeneration after severe herniation injury occurs in neonates and not adults. AF regeneration is mediated by *Scx*-lineage cells that lose *ScxGFP* expression and adopt a stem/progenitor phenotype (Sca-1, days 3–14), proliferate, and then redifferentiate towards type I collagen producing, *ScxGFP*+ annulocytes at day 56. Non *Scx*-lineage cells were also transiently observed during neonatal repair, including *Shh*-lineage cells, macrophages, and myofibroblasts; however, these populations were no longer detected by day 56 when annulocytes redifferentiate. Overall, repair did not occur in adults. These results identify an exciting cellular mechanism of neonatal AF regeneration that is predominantly driven by *Scx*-lineage annulocytes.

## Introduction

Injured adult intervertebral discs (IVDs) heal poorly with the formation of a superficial, fibrous cap at the outermost layers of the annulus fibrosus (AF) and in some cases do not heal at all.^1–3^ Regeneration of the IVD remains an elusive goal due to its complex structure consisting of multiple, developmentally distinct tissues, challenging microenvironment, and continuous mechanical loading at high forces. IVDs are fibrocartilaginous joints that connect adjacent vertebrae and serve primarily mechanical roles to enable spinal motion and support high spinal forces. Important to the IVD’s ability to serve these functions are its two major components, the AF and nucleus pulposus (NP). The AF is a collagen-rich, fibrocartilaginous structure that forms the outer ring of the IVD; the tough, ligament-like AF fibers constrain the centrally-located NP, which is a proteoglycan-rich cartilaginous structure with high swelling propensity. Acute injury or degenerative changes to the AF are commonly associated with IVD herniation, which is defined as extrusion of NP tissue through an AF defect. Herniations can involve radiculopathy (lower back and leg pain and disability) caused by compression and irritation of the NP on adjacent nerve roots. When conservative measures fail to reduce pain and disability from radiculopathy, surgical discectomy is used. In the United States, 300,000–400,000 patients receive discectomy procedures^4^ which treat the acute nerve root compression injury by surgically removing herniation tissue without repairing AF defects. Discectomy has more favorable outcomes than non-operative treatment in most patients, but unrepaired AF defects can contribute to recurrent or persistent radiculopathy, reherniation in up to 27% of patients, and accelerated IVD degeneration.^5–7^ Despite the prevalence of post-discectomy complications, treatment options are limited with varied outcomes and no effective AF repair strategies. Therefore, there is an unmet clinical need for the development of regenerative AF repair strategies that restore IVD structure and mechanical function.

Although adult mammals can efficiently regenerate some tissues such as liver,^8,9^ bone,^10,11^ and muscle,^12,13^ for most tissues (such as heart, pancreas, and nerves^14^), regenerative capacity is absent and the default mode of healing is fibrotic scar formation. Regeneration of normally non-regenerative tissues is therefore typically studied using model organisms (such as axolotls and zebrafish) or more rarely, in specific mouse strains (such as MRL and African spiny mice). Recently, the neonatal mouse has also emerged as an exciting new model of mammalian regeneration that is not limited to a particular mouse strain. To date, the capacity for neonatal regeneration has been demonstrated for diverse tissues including the heart,^15^ cochlea,^16^ digit tip,^17^ tendon,^18^ and IVD.^19^

The adult IVD is normally non-regenerative since AF injury leads to several pathological changes, including decreased IVD cellularity, matrix degeneration, innervation, inflammation, and formation of granulation tissue.^20,21^ Poor healing is generally thought to be a consequence of limited vascularity and the mechanically challenging microenvironment. However, we recently showed that neonatal IVDs are capable of functional regeneration following severe herniation injury, with restored IVD height, biomechanical properties, and improved structural healing compared to adults.^19^ Furthermore, using the transgenic *ScxGFP* reporter, which identifies tenocytes and AF cells (annulocytes),^22^ we observed a population of *ScxGFP-*negative cells occupying the injury site one month after herniation,^19^ suggesting either annulocyte dedifferentiation or recruitment of non *Scx-*lineage (non AF-derived) cells. To address this question, we now identify the cellular players and their dynamics during neonatal and adult AF healing using inducible genetic lineage tracing of *Scx*-lineage (AF-derived, *Scx*-lin) and *Shh*-lineage (NP-derived, *Shh*-lin) cells. We found that neonatal AF regeneration is primarily driven by *Scx*-lin cells that lose *ScxGFP* expression and adopt a stem/progenitor phenotype. Following recruitment, *Scx*-lin cells expand via proliferation and reacquire *ScxGFP* expression and markers consistent with an annulocyte fate. Non *Scx*-lin cells detected during AF regeneration included *Shh*-lin cells, macrophages, and myofibroblasts. However, the presence of these cells is transient. Collectively, these findings identify a key cellular mechanism underlying neonatal AF regeneration and may inform regenerative strategies for improving adult AF healing.

## Results

### Early proliferation and minimal apoptosis following neonatal herniation

**Fig. 1:**
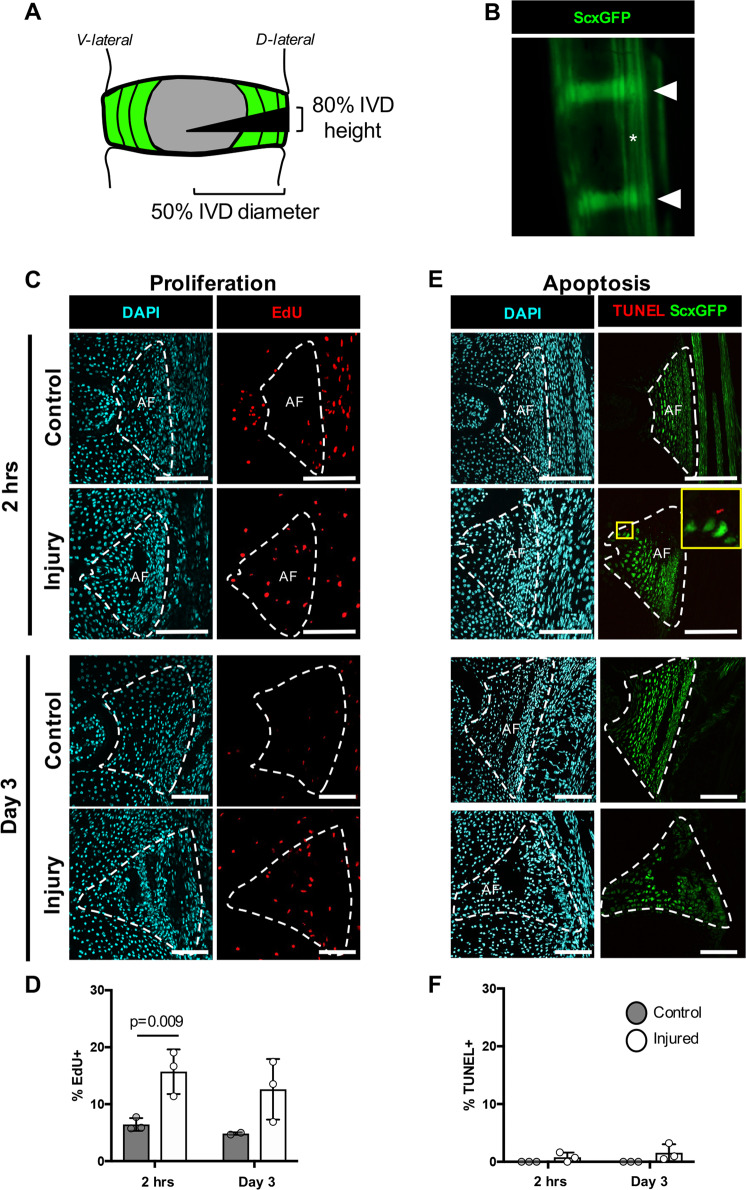
Early proliferation and minimal apoptosis of injury site cells occurs immediately following neonatal herniation. Neonatal injuries were performed using a 31G beveled syringe needle tip to 50% of the IVD diameter **a** in the *ScxGFP* reporter mouse that labels annulocytes (triangles) and tenocytes (asterisks) **b**. *ScxGFP* expression is decreased in the neonatal IVD 2 hrs post-injury (yellow triangle) **b**. Proliferating cells were detected using EdU. Representative images of the posterior control AF of an uninjured IVD and the posterior AF of an injured IVD show an increase in proliferation at 2 hrs and d3 **c**. Quantification using cell counting determined that there was a significant increase in the percentage of proliferating cells at d0 (2 hrs post-injury) compared to controls **d**. Cells undergoing apoptosis were detected using TUNEL staining. Minimal apoptosis was observed in uninjured controls at 2 hrs, where the few cells stained positive for TUNEL were located at the border of *ScxGFP*+ cells and *ScxGFP*- cells in the injured AF and at d3 **e**. No differences between the percentage of TUNEL-positive cells were observed between control and injured AFs **f**. Error bars = SD. Scale = 100 μm.

To establish cellular dynamics in the normal neonatal AF and following neonatal injury, proliferation and apoptosis was determined using EdU and TUNEL detection, respectively. In uninjured animals, annulocytes showed high proliferative capacity during early postnatal stages, with an early proliferative peak observed during the first week of age.^23^ Neonatal IVDs were therefore injured within the first week of age at p5 using the *ScxGFP* reporter to visualize IVD structures directly through the skin. Injury was created using a dorsal-lateral approach with a beveled syringe needle tip inserted to 50% of IVD diameter (Fig. 1a, b). A near-immediate increase in proliferative activity was observed 2 hours (hrs) after injury, with ~14% of cells proliferating in the injured AF, compared to ~8% cell proliferation in uninjured, internal AF controls. Proliferation was maintained at day 3 post-injury (d3), with ~13% of cells proliferating in injury relative to ~7% in controls (Fig. 1c, d). Proliferating cells were also observed in regions near to the injury site, including the growth plates and connective tissue adjacent to the outer AF, at both day 0 and day 3. TUNEL staining showed minimal apoptosis in all samples, regardless of injury or timepoint (Fig. 1e, f). These results suggest that a rapid proliferative response in neonates may be an early driver of regenerative healing.

### Neonatal injury sites are populated by *Scx*-lin and non *Scx*-lin *ScxGFP*+ annulocytes

**Fig. 2:**
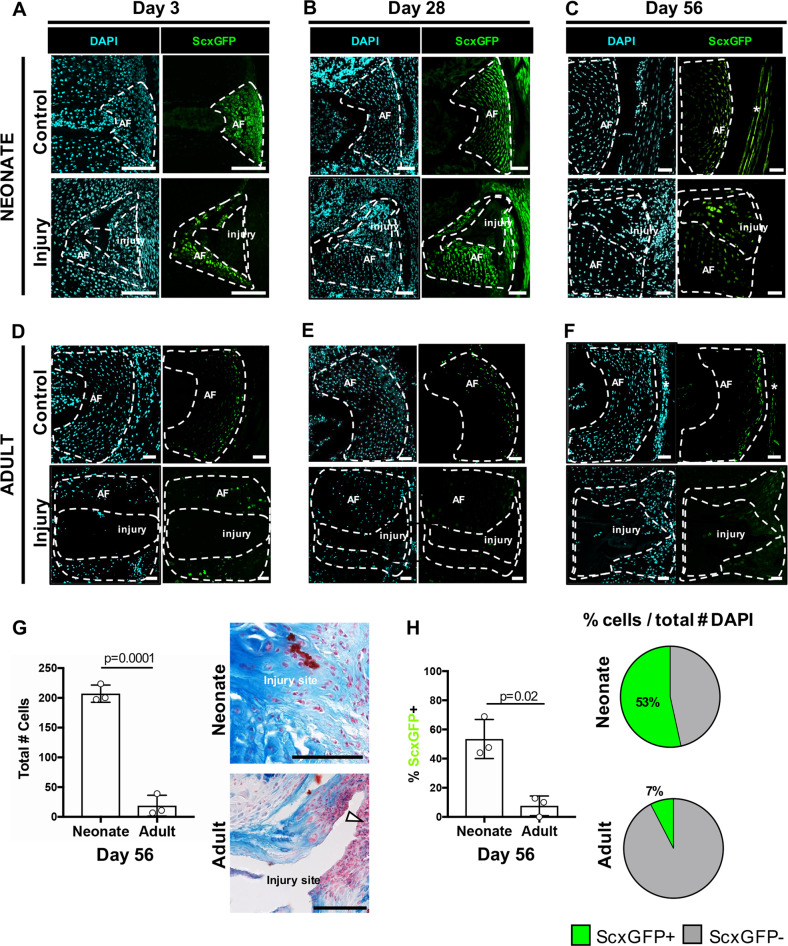
*ScxGFP* annulocyte differentiation occurs in the neonatal injury site by d56. *ScxGFP* expression is observed in the uninjured, control AF in almost all cells at d3 **a**. *ScxGFP* expression is decreased in the inner AF at d28 **b**, and is limited to the outer AF by d56 **c** and at all adult stages **d**–**f**. At d3 following injury, the neonatal injury site is cellular but most cells in the injury site are *ScxGFP*- **a**. At d28, the injury site remains cellular and consists of *ScxGFP-* cells immediately adjacent to *ScxGFP+* cells that appear to be from intact AF and are organized into aligned layers **b**. At d56, *ScxGFP* expression in annulocytes adjacent to the injury site appears decreased, and cells within the injury site express *ScxGFP* **c**. The adult injury site has minimal cellularity at d3, d28, and d56 (**d**–**f**). The neonatal injury site was occupied by extracellular matrix and was highly cellularized, while in adults, the injury site remained largely void of extracellular matrix or cells, and a fibrous, cellular cap lined the periphery of the posterior AF (G, white triangle). The total number of cells recruited to the injury site was increased in neonates compared to adults **g**. *ScxGFP* expression at the d56 is restored in ~53% of cells of the injury site in neonates, compared to ~7% in adults **h**. Adjacent tendon (*). Error bars = SD. Scale = 100 μm.

To determine the dynamics of AF-specific differentiation after injury, *ScxGFP* expression was determined at early (d3), middle (d28), and late (d56) timepoints after neonatal and adult injury. In neonates at d3, the injured AF site was highly cellularized with little *ScxGFP* expression in these cells relative to uninjured controls (Fig. 2a). At d28, the injured AF remained highly populated by *ScxGFP*- cells that were immediately adjacent to *ScxGFP* + cells of the intact AF, identified by its organized lamellar structure (Fig. 2b). At d56, which is when functional biomechanical properties are restored in neonates,^19^ *ScxGFP*+ cells were detected within the injury site, indicative of annulocyte differentiation; however cell density in control and injured IVDs was reduced compared to previous timepoints. Although *ScxGFP* expression in cells of the adjacent, intact AF region was relatively low, this is consistent with low *ScxGFP* expression in the non-injured control AF, in which *ScxGFP* can only be detected in outer annulocytes (Fig. 2c). This downregulation/restriction of *ScxGFP* expression may coincide with the end of AF tissue growth (similar to previous reports in tendon^18^) or downregulation of *ScxGFP* in inner annulocytes, which are more chondrogenic.^24^

In contrast to neonatal recellularization and differentiation of *ScxGFP*+ annulocytes, the injury site of adult AF was largely devoid of extracellular matrix and cells (Fig. 2d–g). Alcian Blue/Fast red staining for cartilage and cell nuclei, respectively, revealed that the neonatal injury site was occupied by repair extracellular matrix and was highly cellular, in contrast to the adult injury site which was occupied by minimal repair tissue and was minimally cellularized, and where healing was limited to a cellular cap at the periphery of the injury site (Fig. 2g). Quantification of the few cells present showed that the percentage of *ScxGFP*+ cells in injured adult IVDs at d56 (7% *ScxGFP+*) was significantly less than in d56 neonates (53% *ScxGFP*+) (Fig. 2h).

Annulocyte differentiation in neonates was further assessed by AF-specific gene expression of *Scx, Tnmd*, *Mkx*, and *Col1a1* at d3 and d56 in uninjured, control IVDs and injured IVDs. In neonates, *Tnmd, Mkx*, and *Col1a1* gene expression were unchanged in injured IVDs compared to controls at d3 or d56, while *Scx* expression was increased at d56 (Supplemental Fig. 1). Tenogenic genes were also unaffected in adults. As expected, *Col2a1* expression was relatively low in all groups, indicating successful removal of NP tissue during dissection and absence of aberrant cartilage differentiation with healing. Scar-associated marker *Fn1* was not affected after injury in both neonates and adults. While it is surprising that AF-specific markers are mostly unchanged overall, it is possible that whole IVD analysis is not sufficiently sensitive to detect changes occurring in the injury site.

### Annulocytes in neonatal regeneration are derived from *Scx*-lin and non *Scx*-lin sources

**Fig. 3:**
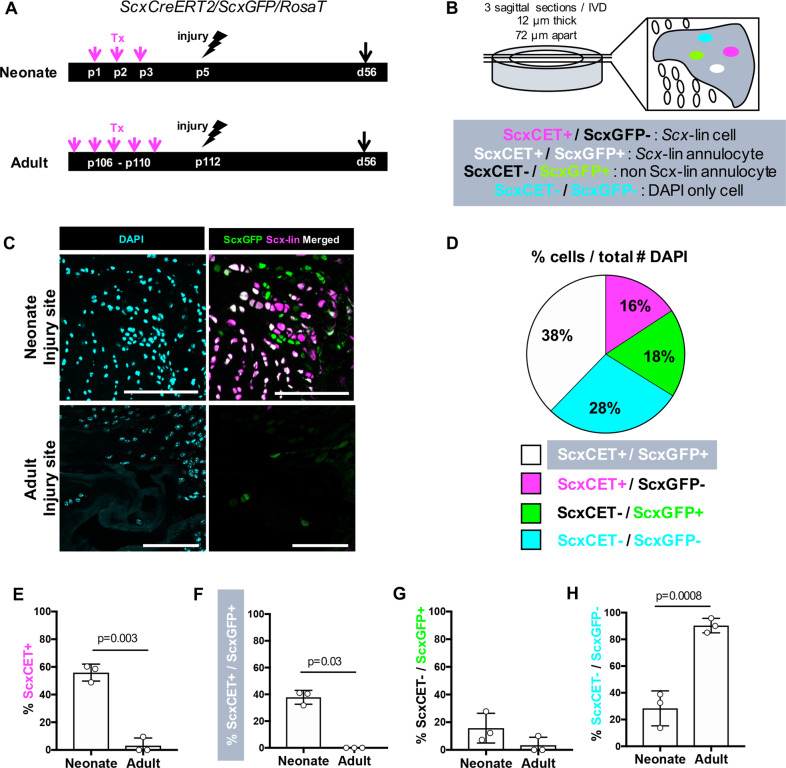
Restoration of *ScxGFP* expression in *Scx-*lin and non *Scx*-lin annulocytes occurs following injury in neonates. *ScxCreERT2/RosaT/ScxGFP* mice were used to trace the fate of annulocytes after herniation in neonates and adults. Annulocytes were labeled by tamoxifen delivery at p1-p3 in neonates or 1 week prior to injury in adults, followed by injury at p5 in neonates and p112 in adults, and subsequent lineage tracing of *Scx*-lin annulocytes at d56 **a**. Four distinct populations of cells were observed in the AF injury site and quantified by averaging three consecutive sections, and include *Scx-*lin cells that are not annulocytes (*ScxCET*+/*Scx*GFP-), *Scx-*lin annulocytes (*ScxCET+/ScxGFP+)*, non *Scx*-lin annulocytes (*ScxCET-*/*ScxGFP+)*, and non *Scx-*lin cells labeled with DAPI only (*ScxCET-*/*ScxGFP-*) **b**. The neonatal injury site was occupied by differentiated *ScxGFP* annulocytes that were also *Scx-*lin cells while the adult injury site was largely devoid of cells **c**. In neonates, the largest population of cells in the injury site were *Scx-*lin, of which 38% were *Scx*-lin (*ScxCET*+) annulocytes and 16% were *Scx-*lin and no longer expressed *ScxGFP*. The next largest population of cells were 18% of cells were neither *Scx-*lineage nor *ScxGFP*+ (DAPI only), followed by the smallest population of cells that were non *Scx-*lin (*ScxCET*-) annulocytes **d**. The percentages of *Scx-*lin annulocytes and non *Scx*-lin annulocytes were significantly decreased in adults compared to neonates at d56 **e**–**g**. The percentage of *ScxCET-/ScxGFP-* cells (DAPI only) in the d56 injury site was significantly greater in adults compared to neonates **h**. Error bars = SD. Scale = 100 μm.

To identify the source of IVD repair cells, we next determined whether differentiation of *ScxGFP*+ annulocytes was mediated by recruitment of *Scx*-lin cells with mitotic potential or non *Scx*-lin cells (potentially stem cells or other wound healing cells). Lineage tracing of *Scx-*lin annulocytes was carried out using *ScxCreERT2* and cells labeled by tamoxifen prior to injury (Fig. 3a). Using *TdTomato* and *ScxGFP* expression, four distinct populations were identified: *Scx*-lin/*ScxGFP*- cells (*Scx-*lin cells), *ScxGFP*+ cells (extrinsically recruited annulocytes), *Scx*-lin/*ScxGFP*+ cells (*Scx*-lin annulocytes), and DAPI only cells (Fig. 3b). Within the neonatal injury site, most recruited cells were *Scx-*lin cells derived from the original AF (~54% *Scx*-lin*+*), and the majority of these *Scx-*lin cells also expressed *ScxGFP+*. A smaller population of *ScxGFP*+ only cells was also observed, derived from non *Scx-*lin sources (~18%, Fig. 3d). By contrast, few cells within the adult injury site were *Scx-*lin (~3%) or *ScxGFP+* (~7%), and significant differences were observed for almost all cell populations relative to neonates (Fig. 3e). *Scx-*lin annulocytes were observed in the neonatal injury site (~40%, Fig. 3f) in contrast to adults where no *Scx-*lin annulocytes were observed. Interestingly, a smaller population of extrinsically recruited annulocytes were observed in neonates (~18%, Fig. 3g), and the few *ScxGFP*+ cells observed in the adult injury site were non *Scx-*lin, although these cells could also be accounted for by incomplete recombination efficiency. Of the few cells observed in the adult injury site, a majority were DAPI only cells (~90%, Fig. 3h) compared to neonates that had a smaller population of DAPI only cells. Together, these findings suggest that recruitment and differentiation of *Scx*-lin annulocytes is a distinctive feature of neonatal AF healing and may drive functional regeneration after injury.

### Loss of *ScxGFP* and proliferation of *Scx*-lineage annulocytes precedes recruitment and redifferentiation

**Fig. 4:**
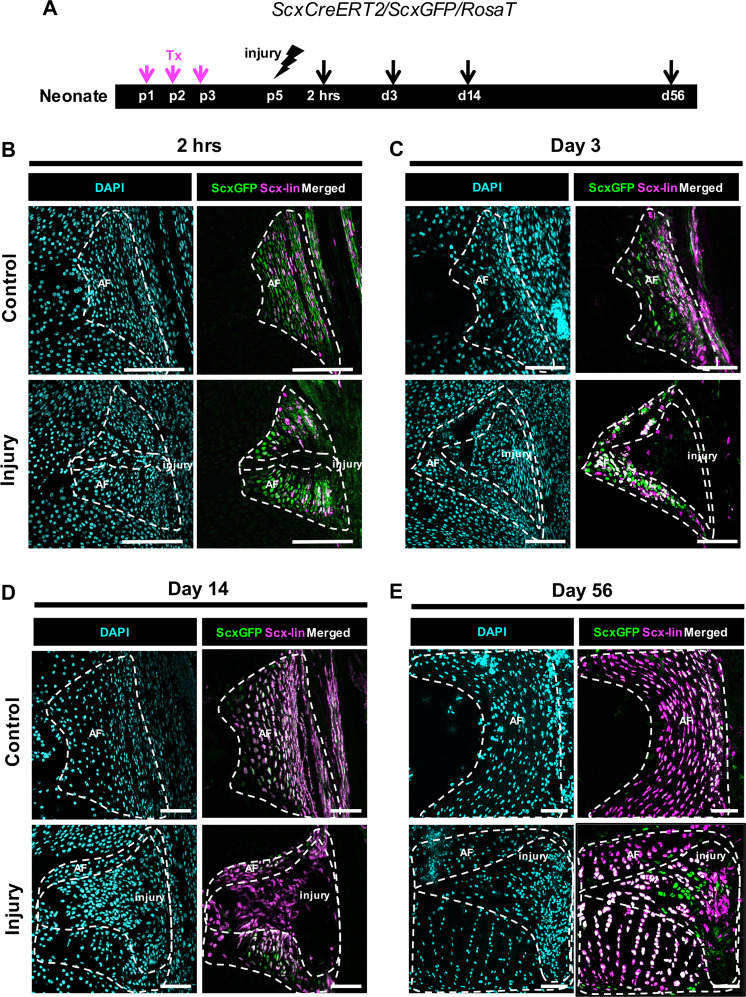
Neonatal AF healing occurs via a transient loss of *ScxGFP* in *Scx-*lin annulocytes followed by restoration of *ScxGFP*. *ScxCreERT2/RosaT/ScxGFP* mice were used to trace the fate of annulocytes that were labeled with tamoxifen at p1-p3, and traced after injury at 2 hrs, d3, d14, and d56 **a**. *Scx*-lin cells in the uninjured control AF were more numerous over time, with a smaller portion of annulocytes labeled at 2 hrs **b** compared to d56 where most annulocytes were *Scx-lin* **e**. At 2 hrs, the injury site was cellular but cells immediately adjacent to the puncture tract lost *ScxGFP* expression and were not *Scx*-lin **b**. At d3, the injury site expanded and contained cells that were neither *ScxGFP+* nor *Scx*-lin **c**. At d14, the injury site remained *ScxGFP-*, and was occupied by *Scx-lin* cells recruited to the injury site **d**. By d56, differentiation of *Scx*-lin cells was observed by co-localization with *ScxGFP* **e**. Images were acquired at 20X and digitally magnified to show the entire posterior AF at each timepoint. Since IVDs are growing at these early stages, the length of the scale bar is decreasing with increasing timepoint. Scale = 100 μm.

To determine the temporal dynamics of *Scx*-lin cell recruitment, annulocytes were labeled at p1-p3 and traced at 2 hrs, d3, d14, and d56 (Fig. 4a). In uninjured control IVDs, all annulocytes were *ScxGFP*+. Labeling of *Scx-*lin annulocytes was incomplete at 2 hrs (Fig. 4b), with improved recombination efficiency at subsequent timepoints (Fig. 4c–e). At 2 hrs, infiltration of DAPI only cells were observed in the puncture tract (Fig. 4b). At d3, the expanded injury site was occupied by a few *Scx*-lin cells, but none of these cells were *ScxGFP*+, suggesting that *Scx*-lin annulocytes had dedifferentiated (Fig. 4c). Increasing numbers of *Scx-*lin cells were observed at d14, although these cells remained *ScxGFP*- (Fig. 4d). By d56, most cells in the injury site were differentiated *ScxGFP+* annulocytes derived from *Scx*-lin and non *Scx*-lin cells (Fig. 4e).

**Fig. 5:**
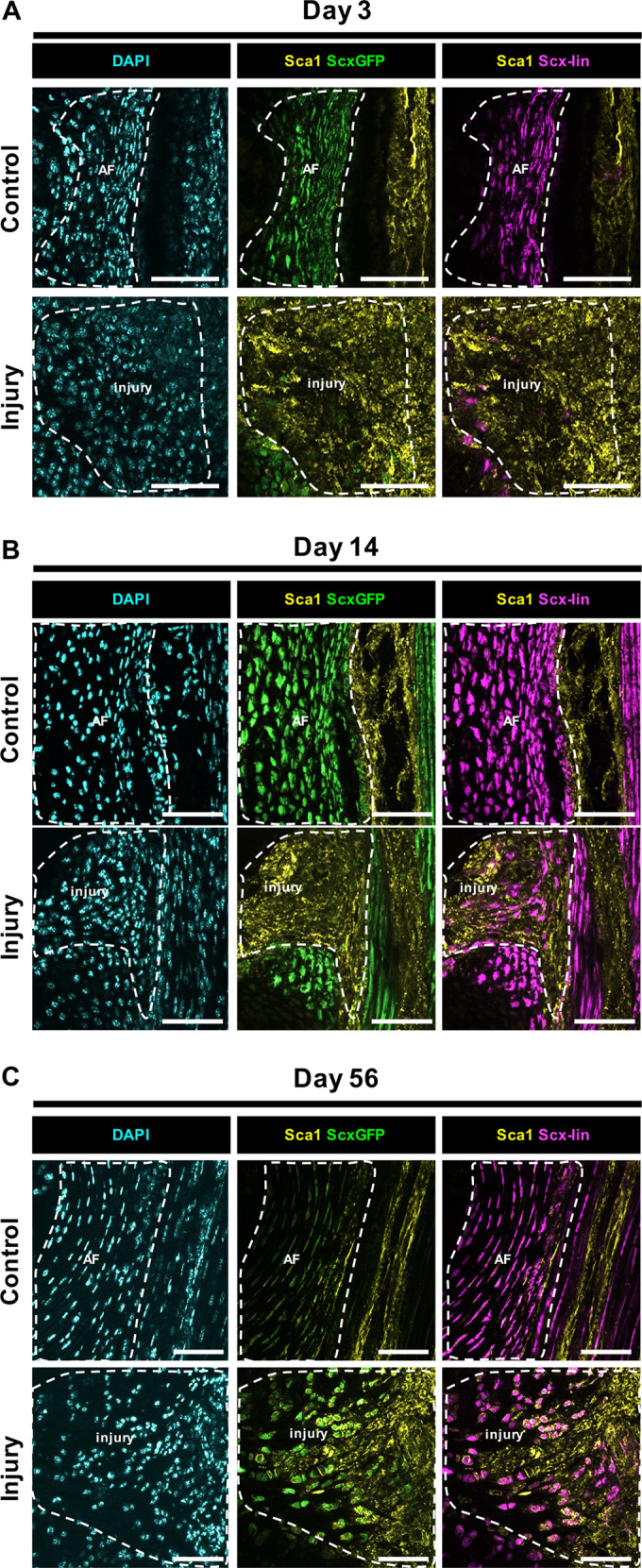
Neonatal *Scx-*lin annulocytes lose *ScxGFP* expression and adopt a stem/progenitor phenotype following injury. Sca-1 immunostaining for stem/progenitor cells at d3 revealed broad presence of non *Scx-*lin cells throughout the injury site and colocalization with few *Scx-*lin cells **a**. *Scx*-lin cells colocalized with Sca-1 at d14 **b** and d56 **c** with decreased Sca-1 expression at the d56 injury site compared to d3 and d14. The presence of non *Scx*-lin, *ScxGFP*+, Sca-1 expressing cells at day 56 points towards contributions of non *Scx*-lin stem/progenitors that are potentially recruited to the injury site and differentiate to a *ScxGFP* cell phenotype. Scale = 100 μm.

To determine whether *Scx-*lin cells observed at the d3 and d14 injury site were potentially dedifferentiated annulocytes adopting a stem/progenitor phenotype, co-localization of stem cell antigen 1 (Sca-1) with *Scx-*lin cells was assessed (Fig. 5). At d3, Sca-1 expressing cells were broadly observed at the injury site. These cells were mostly *ScxGFP-* and the few *Scx-*lin cells present co-localized with Sca-1 (Fig. 5a). At d14, *ScxGFP-* cells occupied the injury site and *Scx-*lin cells co-localized with Sca-1 (Fig. 5b). Cells that expressed Sca-1 but did not co-localize with either *ScxGFP* or *Scx-*lin cells were also observed, potentially indicating recruitment of a stem/progenitor like cell to the injury site. At d56, Sca-1 was present in the injury site but appeared decreased compared to d3 and d14 while *ScxGFP*+ cells were increased (Fig. 5c). Subpopulations of Sca-1 expressing cells were observed at d56 including *Scx-lin/ScxGFP+* cells, non *Scx*-lin/*ScxGFP*+ cells, and non *Scx-lin/ScxGFP-* cells. These findings suggest that *Scx-*lin annulocytes acquire a stem/progenitor like phenotype following injury and points towards contributions from non *Scx-*lin stem/progenitor cells that are recruited to the injury site as early as d3.

**Fig. 6:**
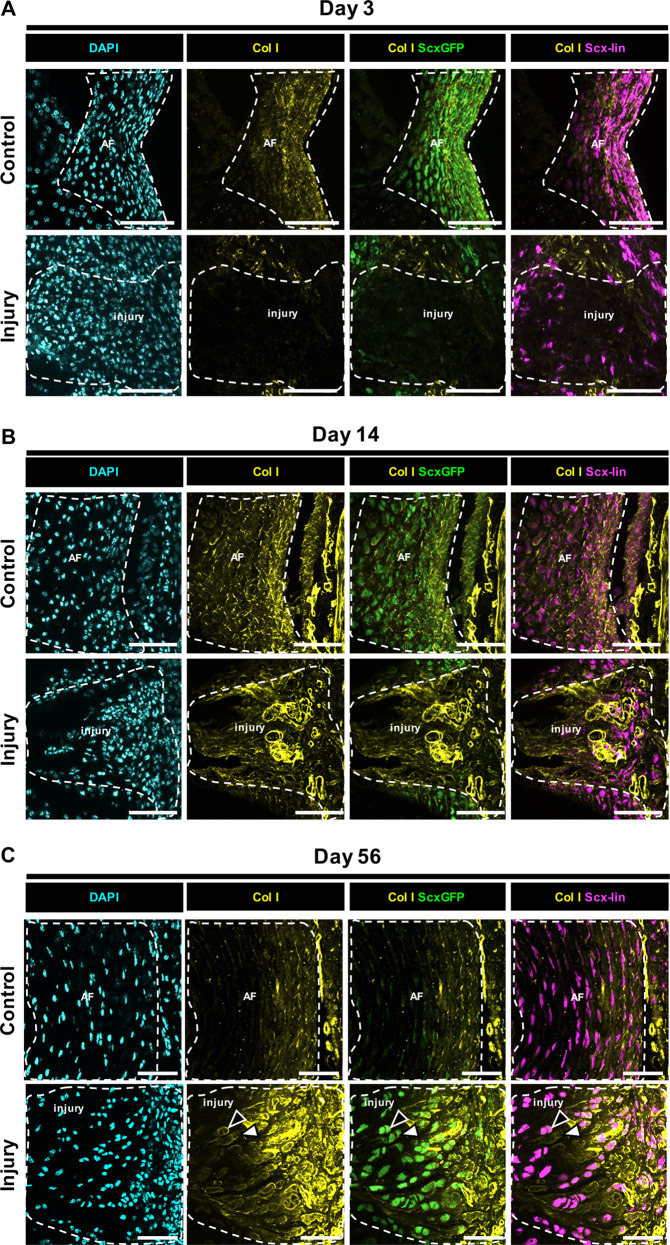
Differentiated *ScxGFP* annulocytes produce type I collagen. Immunostaining for Col I revealed that *Scx-*lin cells in the injury site at d3 were *ScxGFP-* and did not express Col I **a**. At d14, *Scx-*lin cells colocalized with Col I **b**. By d56, *ScxGFP* expression was restored and was colocalized with Col I **c**. Subpopulations of Col I expressing cells were observed at d56 including cells that were *Scx-*lin and *ScxGFP+* (black triangles) and cells that were non *Scx-*lin and *ScxGFP+* (white triangles). *ScxGFP* expression in annulocytes is lost immediately following neonatal injury but annulocyte phenotype is likely restored by d56 as evidenced by colocalization of *ScxGFP* expression and Col I. Scale = 100 μm.

Annulocytes are known to produce type-I collagen (Col I) with a decreasing gradient of expression from the outer AF towards the NP. Col I is therefore a useful marker and the gold standard for determining annulocyte phenotype. Immunostaining for Col I revealed that *Scx-*lin cells in the injury site at d3 were *ScxGFP-* and did not express Col I (Fig. 6a). At d14, *Scx-*lin cells colocalized with Col I (Fig. 6b). By d56, ScxGFP expression was restored and was colocalized with Col I (Fig. 6c). Together, the data suggest that *ScxGFP* expression in annulocytes is lost immediately following neonatal injury but annulocyte phenotype is likely restored by d56 as evidenced by colocalization of *ScxGFP* expression and Col I.

**Fig. 7:**
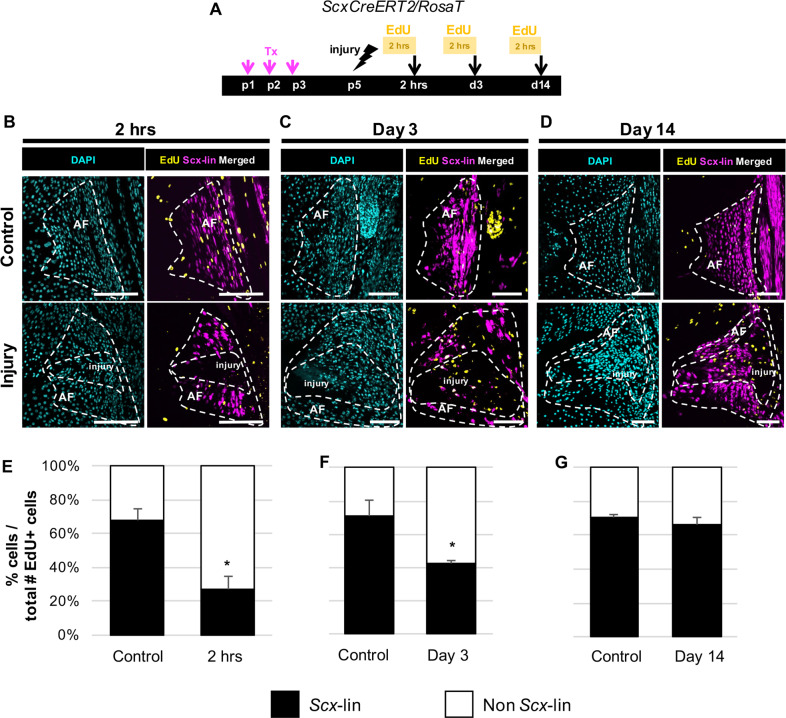
*Scx-*lin annulocyte proliferation contributes to neonatal AF healing. EdU detection of proliferating cells in tamoxifen-labeled controls and injured *ScxCreERT2/RosaT/ScxGFP* mice was performed 2 hrs prior to sac at 2 hrs, d3, and d14 following herniation injury **a**. In uninjured controls, most proliferating cells (~60–70%) are *Scx-*lin cells (EdU+/*Scx*-lin). With injury, the percentage of non *Scx-*lin proliferating cells is increased at 2 hrs and d3 **b**, **c**, **e**, **f**, and is restored compared to control levels at d14 **d**, **g**. Error bars = SD. Scale = 100 μm.

To determine whether expansion of *Scx-*lineage cells observed in the AF injury site was driven by proliferation of dedifferentiated, *Scx-*lin annulocytes or continuous recruitment, EdU was injected 2 hrs prior to harvest (Fig. 7a). In control IVDs, *Scx-*lin cells comprised most proliferating cells at all timepoints (~60–70%, Fig. 7). Although *Scx*-lin cell proliferation decreased 2 hrs after injury relative to controls (Fig. 7b, e), proliferation was improved at d3 and restored by d14 (Fig. 7c–g). At 2 hrs, most proliferating cells reside outside of the injury site, while at d3 and d14, proliferating cells are largely restricted to the injury site. Together, these findings suggest that *Scx-*lin annulocytes dedifferentiate after injury with rapid recruitment into the injury site by d3. Expansion of this population is likely driven by proliferation with redifferentiation by d56.

### *Shh*-lineage cells are transiently recruited to the AF injury site

**Fig. 8:**
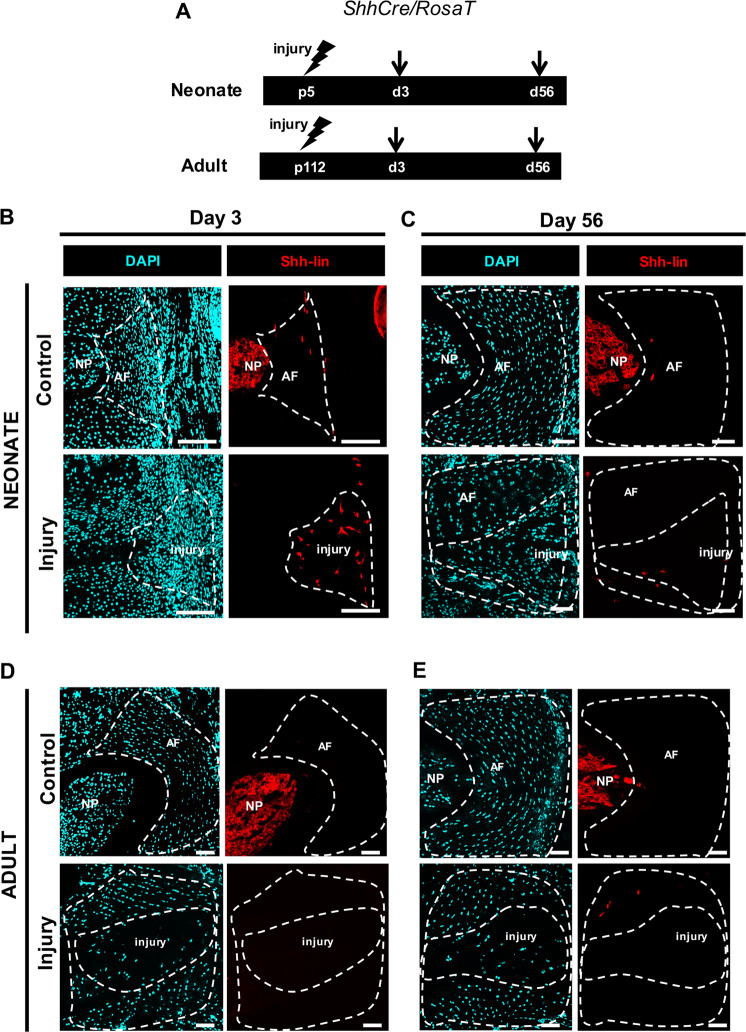
*Shh-*lineage cells are transiently recruited to the AF injury site. Notochordal-derived, nucleus pulposus cells were traced using the constitutive *ShhCre/RosaT* reporter mouse. *Shh-*lin cells were observed in the NP region of uninjured neonatal and adult controls, and sporadically in the uninjured neonatal AF at d3 **b** and d56 **c**, **e**. Early recruitment of *Shh*-lin cells with stellate morphology was observed at the neonatal d3 injury site **b**. Few *Shh*-lin cells were observed in the neonatal d56 injury site **c**. In adults, no contribution of *Shh*-lin cells was observed at d3 **d**, or d56 **e**. Scale = 100 μm.

Although *Scx*-lin cells comprised most cells in the injury site at d56, non *Scx*-lin cells were also observed. One potential source of non *Scx*-lin cells may be herniated NP cells. Therefore, to determine whether NP cells are retained in the injury site or undergo transdifferentiation to annulocytes, lineage tracing was carried out using *ShhCre* (Fig. 8a). Although *ShhCre* is a well-established Cre driver for the NP, we were surprised to observe sporadic *Shh-*lin cells in the AF of uninjured IVDs (Fig. 8b–e). After injury, *Shh*-lin cells were no longer detected in the NP region, consistent with our previous findings indicating complete NP loss following puncture. At d3, a few *Shh*-lin cells with flattened, stellate morphology were observed in the injured AF (Fig. 8b); *Shh-*lin cells were still detected at d56 (Fig. 8c), but the low numbers of these cells suggest minimal contribution of *Shh-*lin cells to regeneration. Immunostaining for the mature NP cell marker cytokeratin 19 (CK19) did not show mature NP cells in the AF injury site (Supplemental Fig. 2). The presence of *Shh*-lin cells may therefore be due to NP cell dedifferentiation, retention of the sporadically labeled *Shh*-lin annulocytes, or *Shh*-lin cell recruitment from a non-NP source. In the adult injury site, *Shh-*lin cells were not detected at any timepoint (Fig. 8d, e).

The stellate morphology of *Shh-*lin cells in the neonatal d3 injury site was not consistent with the typical rounded morphology of NP cells. Since some reports indicate *Shh* signaling may activate myofibroblasts,^25,26^ we immunostained for myofibroblast marker α-SMA in *ShhCre* IVDs. Immunostaining showed no overlap, suggesting that these stellate *Shh*-lin cells are not myofibroblasts (Supplemental Fig. 3). We also screened for other cell types potentially involved in healing, using established markers for macrophages (F4/80), stem/progenitor cells (nucleostemin), pericytes (MCAM/CD146), myofibroblasts (α-SMA), and endothelial cells (PECAM/CD31). Although macrophages and myofibroblasts were transiently recruited to the injury site at early stages, pluripotent stem cells, endothelial cells, and pericytes were not detected (Supplemental Fig. 2). These findings suggest that *Shh-*lin cells, macrophages, and myofibroblasts comprise part of the non *Scx*-lin population during the early phase of neonatal regeneration, however other cells remain unidentified. These results demonstrate a potential role for a population of *Shh*-lin wound healing cells in the early AF injury response.

## Discussion

This study showed that neonatal AF regeneration is mediated by proliferation, recruitment, and restoration of *ScxGFP* and Col-I in *Scx*-lin annulocytes as well as non *Scx*-lin cells that adopt the annulocyte fate. The timecourse for AF differentiation was much longer (56 days) than regeneration of other neonatal tissues (14 or 21 days).^18,27^ This unexpectedly long differentiation process may be due to the mechanically challenging environment and high tissue density, or relatively slow transport and metabolism of the relatively large and avascular IVD relative to other tissues. Nevertheless, our previous findings demonstrate that the neonatal AF regenerates following injury with restored biomechanical function.^19^

Diverse cellular mechanisms have been identified for regenerative tissues; these include transdifferentiation of neighboring support cells (neonatal cochlear hair cells),^16^ differentiation of dedicated stem cell populations (bone, muscle),^28,29^ blastema formation (neonatal digit tip),^17^ and expansion of differentiated cells retaining mitotic potential (neonatal heart, tendon).^15,18^ Here, we find that functional neonatal IVD regeneration is mediated by *Scx*-lin annulocytes with mitotic potential, as well as recruited non *Scx-*lin cells. Proliferation was also observed in regions adjacent to the injury site, consistent with previous studies showing cell proliferation within and adjacent to the injury site in adult salamander and neonatal mouse heart regeneration models.^30,31^ The presence of non *Scx*-lin annulocytes could be the result of incomplete tamoxifen recombination efficiency, but may also indicate a potential contribution from tissue-resident stem/progenitor cells that are activated and differentiate following injury. Similarly, recruited *Scx*-lin cells may be derived from a subpopulation of stem/progenitor annulocytes. Although resident AF stem/progenitor cells have been identified in adult humans and several other species,^32–37^ there is still no consensus in the field on definitive markers for these cells or their location/potential. Although nucleostemin expressing progenitors were previously identified in rabbit AF,^38,39^ we did not observe positive staining in the neonatal mouse IVD. Similarly, CD146+ cells were also not detected. These differences may be due to differences across species or age. Interestingly, Sca-1 was strongly detected in the injured neonatal AF immediately following injury, and restored *Scx*-lin cells appeared to adopt a stem/progenitor phenotype as part of the regenerative healing response. In neonatal controls, Sca-1 cells were observed adjacent to the outer AF and surrounding tendons; these cells may be a source of non *Scx-*lin stem/progenitors observed following neonatal injury. In tendons, contributions of Sca-1 cells migrating into injured tendon from the paratenon may play a role in wound healing responses.^40^ An abundance of *Scx*-lin, Sca-1 expressing cells at the d14 injury site, together with the absence of *ScxGFP* or Col I suggested that some AF cells in the injury site adopted a stem/progenitor state. Further, Sca-1 and *ScxGFP* expression were not mutually exclusive at d56. While this may confirm that *ScxGFP* is not a pure AF differentiation marker (indeed, *ScxGFP* labels tendon and AF progenitors at early embryonic stages^22^), the presence of Col I in the injury site suggests that cells at d56 were observed during a transition stage of differentiation towards an annulocyte phenotype. Sca-1 expression has been used to identify IVD stem/progenitor cells,^41^ but has also been reported in differentiated cell types.^42^ However, the poor regenerative capacity of the adult AF could also suggest that true stem/progenitor cells may not exist for the AF. The identification of robust AF stem/progenitor markers could address this intriguing possibility, enable mechanistic studies that test the regenerative capacity of these cells in vivo, and open novel therapeutic avenues for annulocyte repair. Of note, we employ the term “stem/progenitor” to broadly encompass both stem cells and progenitor cells, which are distinct cell types; stem cells are multipotent and with unlimited self-renewal capacity, and progenitor cells are unipotent and proliferative, with limited self-renewal capacity.^43^ Therefore, in the absence of additional markers to distinguish the two distinct populations, Sca-1 cells observed in this study may fit either criteria.

In this study, we used *ScxGFP* to identify differentiated *ScxGFP* annulocytes and showed that *ScxGFP* expression declines normally with AF maturation and may be associated with impaired adult healing. Although *Scx* is a robust marker for annulocytes from embryonic through postnatal stages,^19,22,44^ *Scx* null mutants do not display a significant AF phenotype,^45^ suggesting it is not required for AF development. Intriguingly, the *Scx* null phenotype is largely limited to long-range tendons throughout the body; short-range tendons, ligaments, and AF are not affected. This suggests that while *Scx* is a consistent marker of all these fibrous cell types, *Scx* function may be highly distinctive between tissues. Elucidating these distinct functions of *Scx* may reveal key developmental processes that distinguish these tissues. In addition to *Scx*, *Sox9* is another interesting marker for annulocytes. All annulocytes originate from the *Sox9*-lineage and conditional deletion of *Sox9* in *Scx*+ cells results in aberrant AF development.^44^ To date, the function of *Scx* and *Sox9* in AF regeneration and healing has not been established. For tendon, *Scx* function is required for matrix deposition after injury but not cell migration.^40^ Identifying the function of these transcription factors in AF healing will be the focus of future studies.

This neonatal AF model of functional regeneration captures key events of reparative regeneration including *ScxGFP* annulocyte differentiation, functional biomechanical restoration, and IVD height recovery. We demonstrated that restored *ScxGFP* cells in neonates colocalized with type I collagen, further supporting the conclusion that these cells may be redifferentiated annulocytes. Interestingly, we noted that Col I signal in the regenerated neonatal AF was markedly more intense than in the uninjured AF. Unmasking using hyaluronidase digestion was required to detect Col I in d56 control IVDs (Supplemental Fig. 5) and it is possible that additional unmasking or enzymatic digestion would be required to address low immunofluorescence signal that was detected in controls. It is also possible that the neonatal injury response induces increased Col I production and remodeling. Interestingly, Col I and α-SMA cells were detected that appeared to form circular structures resembling blood vessels at the intermediate d14 timepoint. However, a marker for endothelial cells (PECAM), which is indicative of vasculature, was not detected. The presence of these structures was transient, suggesting some remodeling occurring at the injury site. However, the hierarchical and lamellar structure of the AF is not restored, suggesting a limitation to neonatal healing. Despite permanent loss of organization within the puncture site, the regions adjacent to the injury site retained its intact lamellar structure and *ScxGFP* expression. This suggests that puncture of neonatal IVD did not trigger the same degenerative cascade that is observed in adults. Developmentally, annulocyte differentiation and alignment precedes collagen fibrillogenesis.^46^ Therefore, in our neonatal model, either *ScxGFP* annulocytes have not realigned by the d56 timepoint, or the signals that drive annulocyte alignment and organization are absent. Since healing is also influenced by the local inflammatory environment,^30,47^ the surrounding matrix, the mechanical microenvironment, and other factors,^23^ it is not surprising that AF regeneration does not fully recapitulate developmental processes.

In contrast to neonates, adult annulocytes were quiescent after injury and minimal proliferation or cell recruitment were observed overall. It is well known that the adult human IVD has limited capacity for endogenous repair and regeneration. This may be due to the loss in proliferative capacity with maturation.^23^ In addition to repair cells that deposit granulation tissue, immune cells such as pro-inflammatory macrophages, T lymphocytes, and mast cells have been implicated in poor adult AF healing.^24,48–51^ However, mechanistic studies testing the requirement of these cells in healing have not been carried out and the sources of fibrotic repair cells remain largely unidentified. Fibronectin is a fibrotic marker associated with IVD degeneration and has been previously identified via immunostaining in degenerated human IVD tissue.^52^ Although we expected increased *Fn1* in adults after injury consistent with our observation of poor adult healing, gene expression results overall were highly variable and we found no changes in *Fn1* expression in neonates or adults after injury. Our inability to detect differences associated with injury and regeneration/degeneration is likely due to the use of the whole IVD for this assay. The use of the whole IVD may not be sensitive enough to reflect histological findings observed at the injury site, which is a small region proportional to the entire IVD. Assessment of AF-specific differentiation and maturation genes at the injury site itself may provide more insight in to the timing of loss of AF phenotype and differentiation, but was considered outside this study’s scope since such localized cell measurements (by laser capture or RNAScope) will require substantial methodological development.

Contrary to the hypothesis that herniated NP cells are retained during neonatal AF healing, *Shh*-lin cells do not significantly contribute to neonatal AF healing. The presence of sporadic *Shh-*lin cells in the control AF was an unexpected finding and has not been previously reported, and is one explanation for the presence of *Shh-*lin cells that were observed in AF regions adjacent to the injury site of some neonatal and adult samples. Labeling of *Shh-*lin cells has been previously observed in the NP and in some cells of the cartilaginous endplate, but not in the AF.^53^ Also unexpectedly, *Shh*-lin cells with non-NP cell morphology were observed at d3 in the neonatal injury site. The constitutive *ShhCre*/*RosaT* mouse labels any cell expressing *ShhCre* with no temporal labeling control, therefore multiple interpretations of these findings are possible. The *Shh*-lin cells observed at the d3 injury site may be an expansion of AF-resident *Shh*-lin cells that were observed in controls, or *Shh-*lin cells recruited from an extrinsic non-NP source. Potential wound healing cells including pericytes were not detected during neonatal healing, however macrophages were observed 2 hrs immediately after injury, indicating a rapid inflammatory response. Macrophages are likely significant mediators of early IVD healing and are required for regeneration in diverse contexts, including neonatal heart regeneration and axolotl limb regeneration.^30,47^ The role of macrophages and the immune response in neonatal AF regeneration warrants further investigation.

One important limitation in our model is the use of a severe herniation injury of healthy IVDs, with percutaneous laceration through several layers of skin, fascia and connective tissue, as well as complete loss of NP. This is less representative of the clinical scenario where herniation is a result of accumulated degenerative damage to the AF and typically does not involve extrusion of the entire NP. It is also possible that the needle puncture itself results in displacement of cells and tissues from surrounding structures into the injury site. However, given that most cells involved in wound healing appeared to be *Scx-*lin, we do not expect that displacement of surrounding cells and tissues resulting from the injury is a primary mechanism driving neonatal healing. Even with this severe injury, the neonatal IVD is remarkably able to restore biomechanical function and *ScxGFP+* annulocytes at the injury site. A more minor injury, which is extremely technically challenging because of the small size of the neonatal mouse IVD, might enable more complete regeneration.

**Fig. 9:**
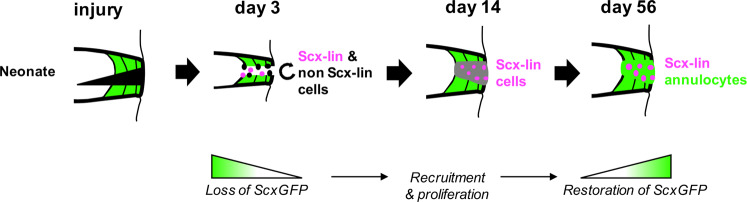
Model of neonatal AF healing. Following neonatal IVD herniation injury, healing was characterized by early activation and proliferation of both *Scx-*lin and non *Scx-*lin annulocytes, some of which are *Shh-*lin. At d14, recruitment of *Scx-*lin annulocytes is observed and their proliferation continues, but cells are not *ScxGFP+* at this intermediate time. By d56, there is differentiation of annulocytes that are mostly *Scx-*lin. This process is mediated by loss of *ScxGFP* expression, recruitment and proliferation of *Scx-*lin cells, and differentiation of *ScxGFP* annulocytes between d14 and d56.

A significant challenge in developing cellular AF repair strategies is the lack of consensus and understanding regarding the cellular composition and cellular phenotypes of cells residing in the AF. The current study contributes to a more thorough understanding of annulocyte subpopulations and regulators of annulocyte differentiation and organization, to help inform choices of appropriate AF cell types and strategies for repair. Here, we demonstrated that functional neonatal AF regeneration is mediated by heterogeneous cell populations that are primarily *Scx*-lin, and that restoration of *ScxGFP+* annulocytes occurs by a series of cellular events including loss of *ScxGFP* expression, proliferation, and redifferentiation (Fig. 9). Our neonatal model of functional AF regeneration holds promise for establishing key cellular populations and molecular signals that regulate annulocyte differentiation and regeneration. Elucidating the biology of regenerative IVD healing will enable novel therapeutic strategies for human IVD repair.

## Methods

### Mice

Existing mouse lines were used in these studies including the *ScxGFP* reporter,^22^ *ScxCreERT2* (generated by Dr. Ronen Schweitzer), *ShhCre* reporter,^54^ and *Ai14 Rosa26-TdTomato Cre* (*RosaT*) reporter.^55^ All transgenic mice are primarily of C57BL/6 background. Lineage tracing of *ScxCreERT2* mice was performed by tamoxifen delivery via gavage in neonates (1.25 mg/pup for 3 consecutive days, followed by 2 days rest) or intraperitoneal injection in adults (100 mg/kg wt for 5 consecutive days followed by 2 days rest) prior to injury. EdU (0.05 mg) was delivered subcutaneously 2 hrs prior to harvest to label proliferating cells. All animal procedures were approved by the Institutional Animal Care and Use Committee and Icahn School of Medicine at Mount Sinai and are consistent with animal care guidelines.

### AF injury model

The neonatal and adult IVD injury models were previously established.^19^ For neonates, IVDs were localized via *ScxGFP* expression visualized under fluorescence directly through the skin with a stereomicroscope with fluorescence capabilities (M165FC; Leica Microsystems), followed by full AF puncture with a 31-gauge beveled syringe needle tip with tissue-marking dye (TBS). Full AF injury was performed to a depth of 50% of the dorsal-lateral – ventral-lateral IVD diameter to induce herniation of NP tissue (Fig. 1a). After injury, neonatal skin was not sutured because of the small size of the injury, and the animals were returned to full cage activity. For adults, IVDs were localized by palpation and exposed by blunt dissection using forceps. A 2–4 mm incision was made in the skin of the dorsolateral aspect of the tail. IVD exposure was visually confirmed under an M60 microscope (Leica Microsystems), followed by full AF puncture with a 26-gauge beveled syringe needle tip, corresponding to 80% of the IVD height (15), with tissue marking dye (TBS). After injury, skin was closed with Prolene 8-0 sutures (Ethicon), and animals returned to full cage activity. Injured and control IVDs were harvested at d0, d3, d14, d28, and d56, for histologic assessment and at d3 and d56, for gene expression studies.

### RNA isolation, reverse transcription, and qRT-PCR

Total RNAs were extracted from uninjured control or injured whole IVDs after neonatal injury using Trizol/chloroform and quantified using NanoDrop 2000. Reverse transcription was carried out using SuperScript VILO Master Mix and qRT-PCR performed using SYBR Green PCR Master Mix (Thermo Fisher). Primer sequences used are listed in Supplemental Table 1. RNA samples were prepared from three injured caudal levels (c4/5, c6/7, c8/9) per animal, with 5–6 animals per group, and RNA from individual IVD levels were run as independent samples in triplicate.

### Histology and immunofluorescence

For histology, neonatal (p5) and adult (p112) mice caudal level (c) 4/5 was injured and c5/6 was used as an internal control with 3 animals per group. For fluorescence imaging and immunofluorescence of sections, tail segments were fixed in 4% paraformaldehyde and frozen in OCT medium. Alternating sagittal cryosections (12 μm) were collected to capture the IVD from outer AF to the opposite outer AF. Immunostaining was carried out using antibodies against Sca-1 (R&D Systems), type I collagen (Abcam), nucleostemin (Neuromics), F4/80 (Affymetrix), α-SMA (Sigma), PECAM (CD31) (BD Pharmingen), MCAM (CD146) (Santa Cruz), and CK19 (Abcam), with DAPI counterstaining (1:1000) to visualize cell nuclei. Primary and secondary dilutions used are listed in Supplemental Table 2. EdU and TUNEL assays were performed using the Click IT EdU and Click IT TUNEL kits (Life Technologies), according to manufacturer’s instructions with appropriate positive controls (Supplemental Fig. 4). All images were acquired using Zeiss Axio Imager microscope; an Apotome was used for optical sectioning of fluorescent images.

### Quantification of cell populations

Three consecutive sections, 72 μm apart, were imaged from the mid-sagittal region, which was determined by the measurer, and the AF and injury site regions were outlined (Fig. 4b). For proliferation and TUNEL measurements at 2 hrs, d3, and d14, the entire posterior AF was quantified. For d56 measurements, only the injury site was quantified. Cell counting was performed in Image J, and the average of three consecutive sections per disc was obtained for each cell population of interest.

### Statistics

All statistical analyses were performed in GraphPad Prism with p < 0.05 significance. One-tailed Student’s t-test determined differences in percentage of proliferating cells and levels of cell apoptosis between control and injury in neonates, differences in the total # of cells recruited to the injury site and percentages of different cell populations between neonates and adults, and percentages of proliferating cells between control and injury in neonates. One-tailed Student’s t-test determined effect of treatment (control and injury) within each timepoint (d3 and d56) on gene expression changes.

### Reporting summary

Further information on research design is available in the Nature Research Reporting Summary linked to this article.

## Supplementary information

Supplemental figures

Reporting summary

## Data Availability

The data that support the findings of this study are available from the corresponding author upon reasonable request.
