# Supplementary material for: Neonatal annulus fibrosus regeneration occurs via recruitment and proliferation of Scleraxis-lineage cells
Source: NPJ Regen Med. 2019 Dec 20;4:23. doi: 10.1038/s41536-019-0085-4 (PMC6925137; doi:10.1038/s41536-019-0085-4)
Supplement: Supplementary file 1 — Supplemental figures [file 41536_2019_85_MOESM1_ESM.pdf]

Supplemental Figure 1: Expression of AF-specific genes during neonatal regeneration. Real time qPCR of AF markers *Scx, Tnmd, Mkx,* and *Col1a1* (A-D), NP marker *Col2a1* (E), and scar-associated marker *Fn1* (F) plotted on a semi-log scale in whole control and injured neonatal and adult AF. Solid line = $p < 0.05$. All p-values (injury vs. control within each timepoint) (G). n = 4–7 IVDs/group.

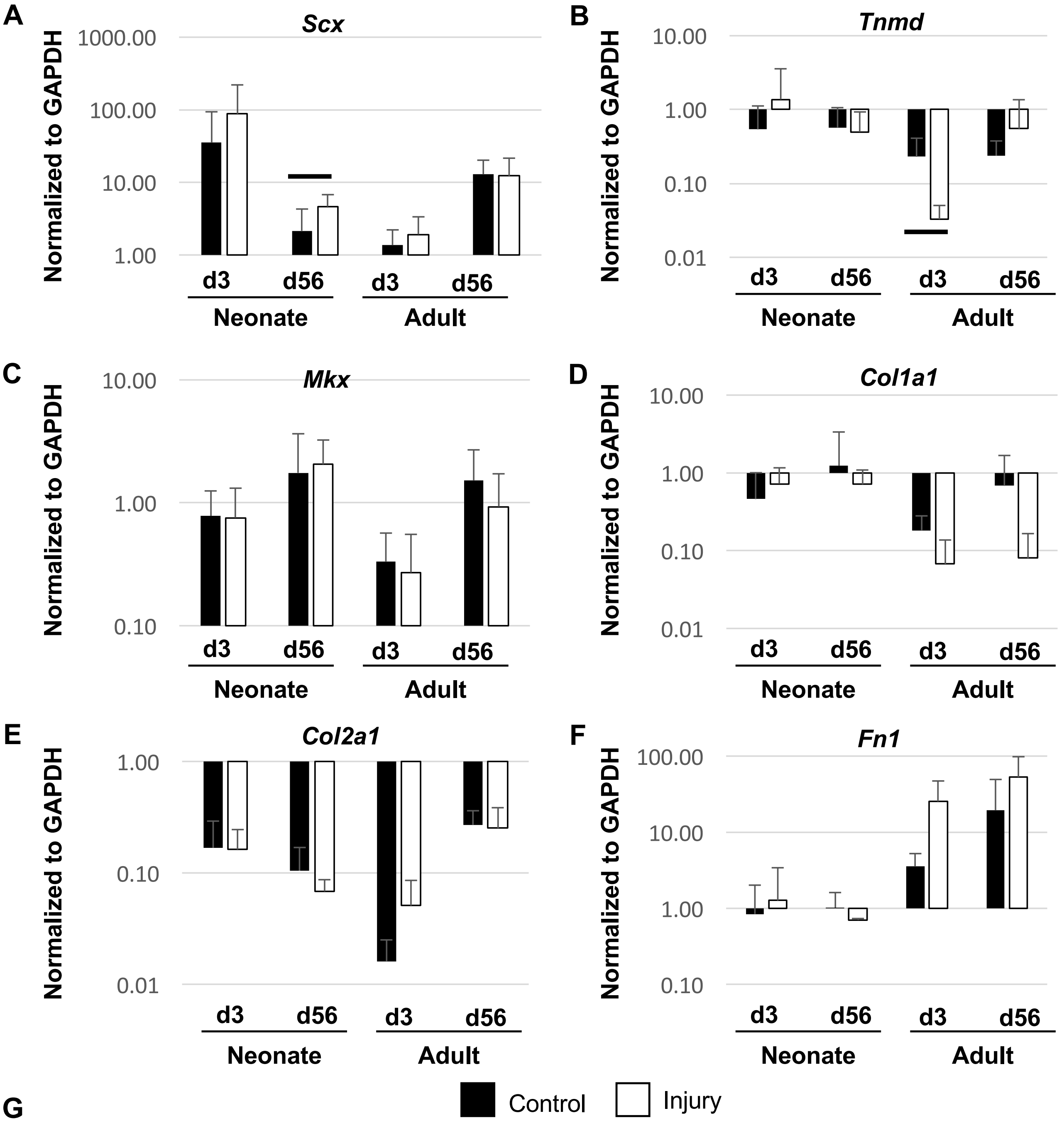

G

	p-values (control vs. injury)					
	Scx	*Tnmd*	*Mkx*	*Col1a1*	*Col2a1*	*Fn1*
Neonate d3	0.11	0.27	0.27	0.23	0.42	0.39
Neonate d56	**0.0015***	0.43	0.36	0.34	0.32	0.28
Adult d3	0.10	**0.05***	0.37	0.13	0.13	0.07
Adult d56	0.42	0.21	0.22	0.22	0.30	0.19

Supplemental Figure 2: Potential cell types involved in neonatal wound healing. Pluripotent stem cells (nucleostemin) were not observed in the injury site at day 0 (A). Macrophages (F4/80) were observed in the injury site at day 0 (B). Myofibroblasts (α-SMA) were observed at day 14 (C), and did not co-localize with an endothelial cell marker (PECAM) (D). Pericytes (CD146) and NP cells (CK19) were not observed at day 14 (E, F). Scale = 100 μm.

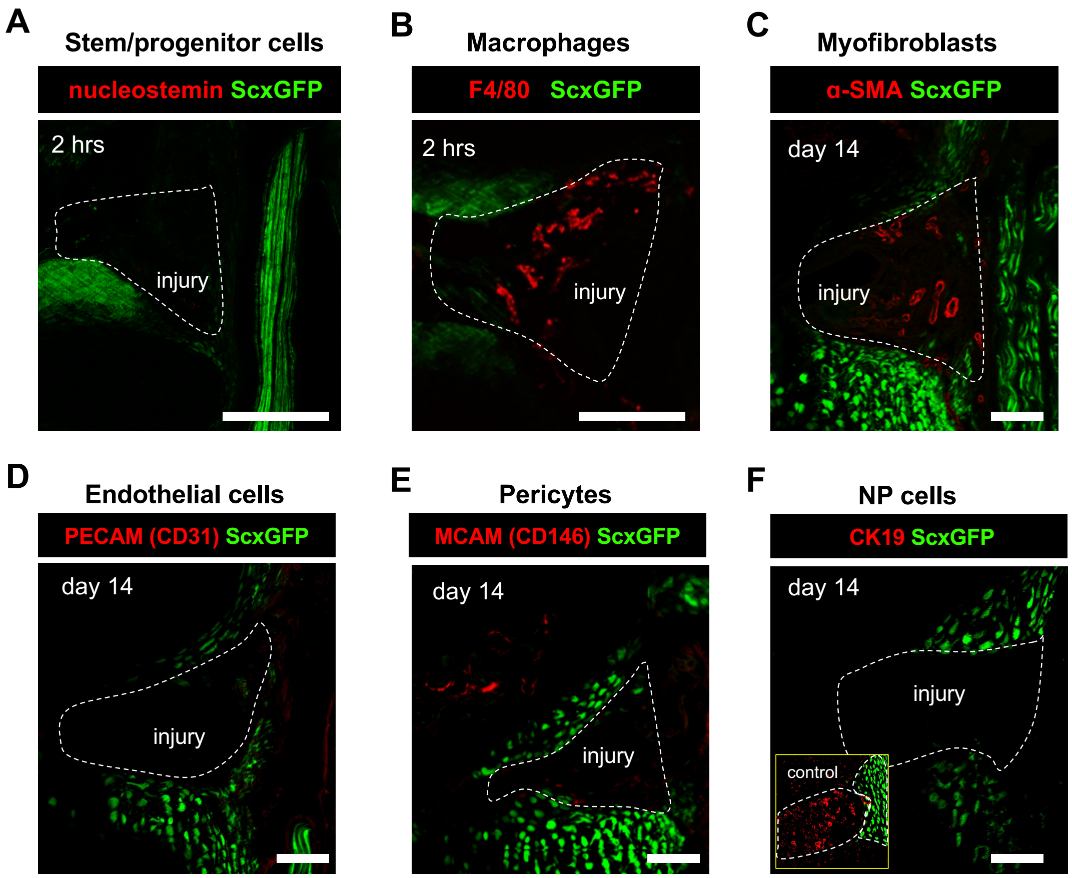

Supplemental Figure 3: Early *ShhCre* cells at the AF day 3 injury site are not myofibroblasts. Scale = 100 µm.

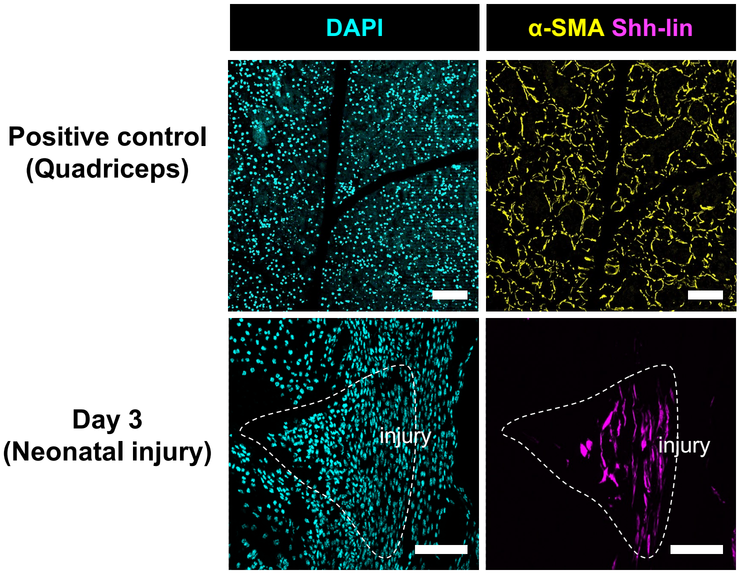

Supplemental Figure 4: AF cell proliferation in uninjured, control IVDs during early postnatal growth. Scale = 100 μm.

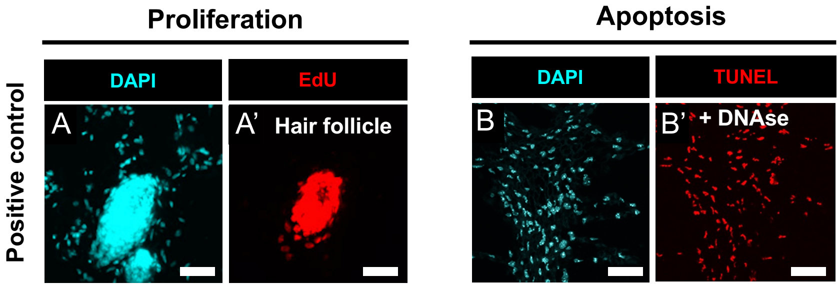

Supplemental Figure 5: Different Anti-Col I immunostaining protocols affect antibody signal detection. Anti-Col I was applied using a 1:400 dilution (secondary: Cy5, 1:400). Staining using 0.05% Triton-X for all wash steps resulted in diffuse staining of adjacent tendons, and staining of only the outermost AF. Histozyme antigen retrieval solution was applied for 10 min at room temperature prior to immunostaining, with similar results (A). To reduce diffuse signal and improve localization of Col-I signal to the pericellular region, 0.05% Triton-X in 1X PBS was only applied immediately prior to the secondary and resulted in more localized signal in tendons and the outermost AF layer (B). To address potential masking effects of proteoglycans in the AF, 2mg/mL hyaluronidase in 1X PBS (pH 5) was applied for 30 min at 37°C prior to immunostaining, and resulted in improved Col I detection in the outer AF with a gradient of signal intensity from the outer AF towards the NP (C). Scale = 100 μm.

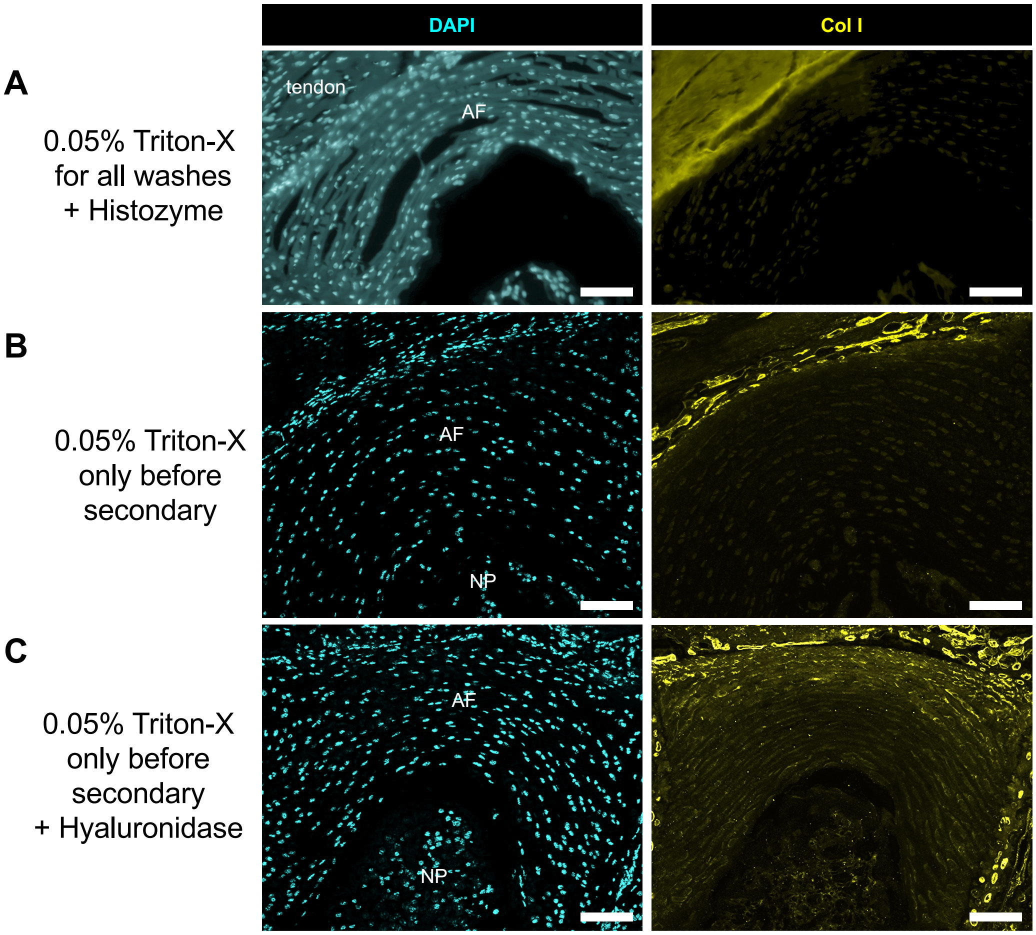

Supplemental Table 1: qPCR primers used for whole IVD gene expression.

Primer	Forward Sequence	Reverse Sequence
Scx	CGTCTTTCTGTCACGGTCTTTGCTC	CTTTCTTCCACAGCGGTCGTGC
Tnmd	GGGCTGTCACATTCTAAATGCAG	TTCTTCTTCTCGCCGTTGCT
Mkx	CGTGACAACCCGTACCCTAC	TTTGACACCTGCACTAGCGT
Col1a1	ACGCCATCAAGGTCTACTGC	ACTCGAACGGGAATCCATCG
GAPDH	CCATGACAACTTTGGCATTG	CCTGCTTCACCACCTTCTTG

Supplemental Table 2: Primary and secondary antibodies used for immunostaining of IVD cryo-sections.

	Antibody	Vendor	Host	Dilution
1°	anti-Sca1	R&D Systems	Goat	10 µg/mL
	anti-Collagen I	Abcam	Rabbit	1:400
	anti-nucleostemin	Neuromics	Goat	1:100
	anti-F4/80	Affymetrix	Rat	1:500
	anti-actin, alpha-Smooth Muscle	Sigma	Mouse	1:100
	anti-PECAM (CD31)	Abcam	Mouse	1:100
	anti-MCAM (CD146)	Santa Cruz	Mouse	1:25
	anti-CK19	Abcam	Rabbit	1:200
2°	anti-goat (Cy3)	Jackson ImmunoResearch	Donkey	1:400
	anti-rat (Cy3)	Jackson ImmunoResearch	Donkey	1:400
	anti-mouse (Cy3)	Jackson ImmunoResearch	Donkey	1:200
	anti-rabbit (Cy3)	Jackson ImmunoResearch	Donkey	1:400
